# Using Amino Acid Correlation and Community Detection Algorithms to Identify Functional Determinants in Protein Families

**DOI:** 10.1371/journal.pone.0027786

**Published:** 2011-12-20

**Authors:** Lucas Bleicher, Ney Lemke, Richard Charles Garratt

**Affiliations:** 1 Departamento de Bioquímica e Imunologia, Instituto de Ciências Biológicas, Universidade Federal de Minas Gerais, Belo Horizonte, Minas Gerais, Brazil; 2 Departamento de Física e Biofísica, Universidade Estadual Paulista, Botucatu, São Paulo, Brazil; 3 Departamento de Física e Informática, Instituto de Física de São Carlos, Universidade de São Paulo, São Carlos, São Paulo, Brazil; British Columbia Centre for Excellence in HIV/AIDS, Canada

## Abstract

Correlated mutation analysis has a long history of interesting applications, mostly in the detection of contact pairs in protein structures. Based on previous observations that, if properly assessed, amino acid correlation data can also provide insights about functional sub-classes in a protein family, we provide a complete framework devoted to this purpose. An amino acid specific correlation measure is proposed, which can be used to build networks summarizing all correlation and anti-correlation patterns in a protein family. These networks can be submitted to community structure detection algorithms, resulting in subsets of correlated amino acids which can be further assessed by specific parameters and procedures that provide insight into the relationship between different communities, the individual importance of community members and the adherence of a given amino acid sequence to a given community. By applying this framework to three protein families with contrasting characteristics (the Fe/Mn-superoxide dismutases, the peroxidase-catalase family and the C-type lysozyme/α-lactalbumin family), we show how our method and the proposed parameters and procedures are related to biological characteristics observed in these protein families, highlighting their potential use in protein characterization and gene annotation.

## Introduction

### Overview of amino acid correlation methods

The observation of patterns of correlation at certain positions in a protein family multiple sequence alignment has been described at least since the eighties. It gained considerable attention in the nineties, when a variety of correlation metrics were proposed. Since then, the primordial application of correlated mutations has been the search for contact pairs - indeed it was soon observed that two positions showing strong correlation would probably be near in the protein three dimensional structure, as proposed in Göbel's seminal article in 1994 [1].

The search for sets of co-evolving position has also been discussed since the late 90's. Atchley, et al. [2] used a simple procedure to find “cliques” of co-evolving positions in a quantity named “position association” (“pa-values”), an estimation of their mutual information. Cliques were defined as groups of positions such that any two positions within one have pa-values among the highest 5% of all such values [2]. In the same year, a different metric of positional correlation was proposed by Lockless and Ranganathan [3]. Loosely based on Boltzmann's statistical mechanics, their “statistical coupling analysis” (SCA) presented two energy-like parameters to quantify both positional conservation and inter-positional correlation. The first one, termed ΔG, measures the overall conservation in a alignment position and correlates well with the well known measure known as sequence entropy [4]. The second one, termed ΔΔG, measures the effect, in an alignment position, of having a given amino acid in another position. This procedure is called a “perturbation”: therefore, ΔΔG_i|j = ALA_ will measure how much the distribution of amino acids at position i varies when there is an alanine at position j. This metric has two interesting features. The first one is the “perturbation” concept, which results in much more useful information when measuring the correlation between positions. Being able to saying that, for a given alignment, position 25 and 45 are highly correlated is much less informative than saying that, when there's a cysteine at position 25, the fraction of cysteines at position 45 increases considerably. While the former only suggests that the two positions may be in contact in the three dimensional structure, the latter also suggests that there might be a disulfide bridge connecting the two positions for a subset of the proteins - those having a cysteine at both positions. Although it was possible to derive this information using SCA, the authors only reported the overall ΔΔG between two positions given a particular amino acid at one of those positions. For example, the method only reported that the presence of a cysteine at position 25 resulted in a large variation of the amino acid distribution in position 45 when compared to the overall alignment. Furthermore, the authors have dropped this metric in more recent articles, using instead a positional correlation method which abandons the “perturbation” concept, only informing how much the two positions are correlated [5], much like other correlation metrics which already existed. Another interesting property of the original SCA correlation metric is the fact that, since it uses binomial probabilities, it automatically takes into account the sample size. So, if a given correlation happened for 500 sequences in a 1000 sequence alignment, its measure is much bigger than the same correlation for 50 sequences in a 100 sequence alignment (something that would not happen for mutual information, for example). However, it also has serious drawbacks. The idea of an energy-like parameter was used in order to “measure energetic coupling between positions on a multiple sequence alignment” [3], and was supposed to be a theoretical alternative to experiments such as thermodynamic cycle analysis. Although initially it seemed that there was a linear correlation between the two quantities for the selected residue pairs, subsequent studies showed that correlated mutation algorithms (including SCA) can find residue pairs which are close to each other and that these tend to be thermodinamically coupled, but there is little evidence that thermodynamic coupling is limited to residue pairs obtained by these methods [6], [7].

Dekker and co-workers [8] proposed a perturbation-based method which, instead of using the energy-like approach, was based in calculating the explicit likelihood of the observed covariances, therefore providing a measure which is more directly related to co-variation statistics [8], instead of an energy-like quantity with no actual connection to a real energy. It still measured the effect of a given “perturbation” (the presence of a given amino acid in a position) on the overall distribution of another site, but it showed increased predictive power in finding native contacts, when compared to the original SCA algorithm. It was also still dependent on a method to determine what would be the “smallest significant perturbation”, and the authors used the empirical jackknife-like procedure described by the developers of SCA [9]. A theoretically sound procedure was proposed by Dima and Thirumalai, based on choosing the smallest sub-alignment that would still satisfy the central limit theorem [10]. This procedure would reduce spurious results which would arise if poorly conserved columns are included in the analysis.

From 2003 onwards [9], correlation (ΔΔG) matrices were subjected to clustering methods in order to obtain a set of self-correlated positions. These sets were postulated to represent the “structural motifs for allosteric communication in proteins” [9]. However, little attention has been paid to the fact that sets of correlated (or anti-correlated, since both correlation and anti-correlation implicate in positive ΔΔG values) positions can have very different meanings which could be better understood by analyzing the individual contributions from each amino acid type, the topology of the generated network, and the differentiation from correlation and anti-correlation.

In recent work [11], we observed that correlated positions could cluster into different groups, related to different properties in a protein family, the Fe/Mn superoxide dismutases (SODs). Members of the family can be either dimeric or tetrameric, and usually selectively bind either Fe or Mn at the active site in a non-substitutable fashion in order to present catalytic activity. The clusters found seemed to be related to positions which were already described as determinants of oligomeric state and metal selectivity [12]. Therefore, instead of the previously postulated view that clusters of correlated positions would reflect routes of allosteric communication or energetic coupling, a much more reasonable hypothesis can be proposed from these results: if a given protein family is populated by proteins having distinct properties (e.g., binding or not a given ligand, having different oligomeric states, being able to interact or not with a given protein, etc.), it is expected that these properties may not be determined by the presence of a single amino acid, but rather a group of them – and this group will emerge from a correlation analysis if a sufficient number of proteins present those properties. However, it may be common to have cases when these residues overlap in which case using any overall positional correlation metric would only result in finding that *there is a set of highly correlated positions*, but not differentiating complementary classes whose key residues are in the same positions (as would be the case in binding site selectivity, for instance).

By measuring specific positional correlation between pairs of residues in given positions, it is possible to define a network in which the existence of a connection between two nodes (nodes being given amino acids in specific positions, say, H34 and S52) implies that sequences having the first amino acid in that position also tend to have the second one in the other position. By defining such a network, it is possible to use algorithms for the detection of community structures. A community in a network can be defined as a group of nodes showing strong connections between them, but not to the rest of the network. This topic has been receiving considerable attention by different groups since the early 2000's [13], [14], [15], and is now applied to a large variety of problems from computer to social networks, and also to the life sciences, especially in the field of molecular systems biology.

In order to tackle the problem of detecting and assessing communities in a network, it is necessary to have a measure of community structure. This can be done using the definition of *modularity* [14], which is a quantity related to a network and a division of that network into groups. If there is a high number of edges (connections) between the vertices (nodes) of the same group, but not many between vertices from different groups, the resulting modularity will have a high value. Community detection algorithms, therefore, engage in maximizing modularity, either by brute-force search or by heuristic methods, which may be needed for large networks. The original definition of modularity can be expanded to include *weighted edges*, i.e., by specifying the strength of the link between the two nodes, *directed edges*, for cases when the connection from A to B is not the same as from B to A, and, finally, *negative weights*, to reflect the fact there may be cases in which it is needed to represent the fact that two nodes *should not* be connected. This is particularly useful for correlated mutation studies, since, at times, there is *anti-correlation* between pairs (e.g.: having an alanine in position 54 results in having much fewer serines in position 78 than expected). A generalization of modularity to include those cases is described in [16].

In this article, we propose a simple method which exploits and quantifies specific correlations. Then we show how networks built from these quantities can be analyzed by community detection algorithms in order to find groups of specific amino acids which tend to be present simultaneously. Finally, it is shown how these results can be used to provide determinants for properties in a given protein family by testing the method for three families with distinct characteristics. The first, Fe/Mn-SODs, is the “ideal case”: they are very well distributed in nature, with almost four thousand sequences available in 1629 species, and they have two independent properties (metal specificity and oligomeric state) which are known for various members of the family. The second is the peroxidase-catalase superfamily, which can be subdivided in three classes with distinct characteristics, but for which the high number of correlated pairs imposes a challenge for the community detection procedure. Finally, the third family, C-type lysozyme/alpha-lactalbumin, also presents two characteristics which can be readily tested (lysozyme activity and calcium binding), but since they are present only in metazoan, with 693 total sequences spread among 254 species, sampling is much more limited.

### Theory

Since we are interested in obtaining a method to explore the specific interdependence of amino acids in given positions, the most naïve approach would be to define that amino acid *x* at position *i* and amino acid *y* at position *j* are correlated when every sequence presenting *x* in *i* also presents *y* in *j*. This has a series of problems which need to be resolved. First, we need to address the fact that there is noise and uncommon sequences in the alignment which would result in the above criterion being accepted only very rarely. Changing it to “having *x* in *i* results in having *y* in *j* for at least 85% of the cases” is slightly better, but would still lead to spurious results: if there are only a very limited number of sequences having *x* in *i*, as the result would be statistically insignificant, and also if virtually all sequences have *x* in *i*, these two positions are not correlated, but rather, strictly conserved. So, the putative correlated pairs must be filtered not only by a minimum frequency, but also by a quantity which measures the significance of the frequency shift for *y* in *j* upon the presence of *x* in *i*.

Suppose that, in an alignment of N sequences, there are n_A_ sequences with a given amino acid *x* at position i and n_B_ sequences with a given amino *y* acid at position j. We want to test whether the presence of *x* in position *i* has any correlation to the presence of *y* in *j*. If they were uncorrelated, the observed frequency of *y* in *j* for the subset of sequences having amino acid *x* in position *i* would still be n_b_/N, which is our null hypothesis. If it is not, we measure the corresponding p-value using the cumulative binomial distribution cbd(N,n,f) as described:

The expected number of sequences having *y* in *j* for the subset having *x* in *i*, considering no correlation, is n_A_(n_b_/N). If the observed number, n_B|A_, is greater than this, we need to measure the probability of observing at least n_B|A_ occurrences in n_A_ trials, of an event whose probability is n_b_/N, i.e.:
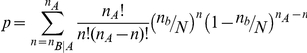



If, conversely, the observed number of residues *y* in position *j* is less than expected, we use the opposite tail of the cumulative binomial distribution to measure the probability of having no more than this observed value, i.e.:
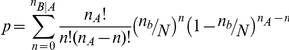



Therefore, we can use −log(p) as a measure of the correlation between two positions. Using log(p) instead of −log(p) in the second case, it is possible to denote anti-correlation.

There are two known sources of bias which need to be addressed. The first is the size of the perturbation, that is, the minimum number of sequences having a given amino acid in a certain position in order to measure its effect. This has been addressed by some authors, and we have adopted the approach of Dima and Thirumalai, based on the satisfaction of the central limit theorem [10]. Finally, another source of bias is the variety of the multiple sequence alignment. If half of an alignment is populated with identical sequences, for example, every pair of amino acids appearing in that repeated sequence would be measured as very highly correlated. Although this example describes a very unlikely situation, it is known that the protein databases are populated by mutations and polymorphisms, and also the distribution of organisms having sequenced genomes is not well balanced among the branches of the phylogenetical tree, therefore this effect must be taken in consideration. While one can imagine different approaches to exploit this feature, since this article deals only with the extraction of overall correlations in a protein family which can be used to detect and characterize classes/sub-families, we simply apply an identity-based culling procedure: if two sequences have more than a given pairwise sequence identity, one of them is removed from the database. This procedure aims to remove possible local correlations that would arise due to very similar sequences, while still maintaining those that are well spread over more distant taxa.

Networks of residue correlations are built using individual combinations of residue type plus position as vertices, and −logP as the edge weight between two nodes. Pairs presenting anti-correlation have reversed sign. In order to restrict to meaningful correlations, the possible pairs are filtered simultaneously by p-value and frequency thresholds called minlogp and Δf: Using minlogp = 10 and Δf = 0.15, for example, means that a pair of vertices remain connected if the presence of the first increases the frequency of the second to more than 85% and vice versa (or decreases to less than 15%, when they are anti-correlated) and the p-value for this frequency shift is less than 10^−10^.

The network can then be submitted to a community detection procedure, for which there are currently many algorithms. Here, we use the community detection by maximization of modularity [14] for the most general case [16], [17], [18], using taboo search plus fine tuning [19], [20] as heuristics.

Communities can be assessed by “self correlation matrices”, as in Table 1, in order to observe why these nodes form a community. They are useful to check for “outliers”, i.e., members with insignificant or negative correlation with the remaining members, which were included simply because, at the end of the community detection procedure, all nodes are compulsorily placed in a community.

**Table 1:** Self-correlation matrix for community 1 in Fe/Mn superoxide dismutases.

POS	ALL	D146	G71	G72	H73	M25	Q145
**D146**	54.1	X	78.4	73.1	66.7	81.7	89.7
**G71**	60.4	87.7	X	86.1	81.4	93.3	99.7
**G72**	63.9	86.3	90.9	X	89.1	97.7	99.4
**H73**	57.3	70.7	77.2	80.0	X	85.8	79.4
**M25**	51.1	77.3	78.9	78.2	76.5	X	87.7
**Q145**	51.7	85.8	85.3	80.5	71.6	88.7	X

Column *all* refers to the overall frequency for the first column (e.g., H is present at position 73 in 57.3% of the sequences). Subsequent columns refer to the frequency *upon* the presence of the given amino acid residue in each column (e.g., the presence of a glycine in position 72 raises the rate of glutamines in position 145 from 51.7% to 80.5%).

We can also define some quantities and procedures in order to analyze the community structure in more detail.

Given that V(A) = [a_1_,a_2_,…,a_N_] and V(B) = [b_1_,b_2_,…,b_M_] are the vertices in communities A and B, respectively, and w(a_n_b_m_) is the weight of the edge connecting a_n_ and b_m_ (*−log(p)* or *log(p)*, depending if there is correlation or anti-correlation), we can calculate the quantity Δ_AB_, shown below, to compare two communities:
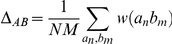



If Δ_AB_ approaches zero, then communities A and B may be related to functions which are independent of each other. If it is large and negative, then there might be characters represented by the amino acids present in the communities which are mutually exclusive. If it is large and positive, then they are correlated - which may be a rare case to find since, if the two communities are correlated, they would probably be merged into one by the community detection algorithm. However, if the edge weights between the two communities are positive but below the significance threshold used (minlogp), they will not be merged during the community detection phase. Also, if A = B, this parameter represents the overall connectivity within a single community, and so could be used to rank communities from the most significant to those with less correlated members.

The total number of community members is affected by variables such as alignment size and the −log(P) cutoff to include pairs of vertices. This is an undesirable feature - if, for example, one wants to plan site-directed mutagenesis experiments in order to see how the amino acids found affect the property which is putatively associated to a community, which residue should first be mutated? Therefore, it is necessary to define a procedure to rank the community members to find those which would arise in most settings. A simple iterative procedure to order the members by “importance” for the community is shown below:

1. Calculate the sum of the correlation scores for each community member against all others.
2. Remove the member which results in the lowest sum (meaning that the “connectivity” of this member to other members is lower and its removal implies the least overall connectivity between remaining members).
3. If there are more than two members in community A, go to 1.

The members will be removed in ascending importance for the community structure, with the two most important (expected to be highly positively correlated) left at the end of the procedure.

We can also define an adherence measure to quantify the extent to which a given sequence fits into a given community.
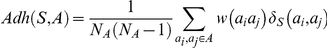



The delta function δ_S_(a_i_,a_j_) takes the value 1 if both vertices (i.e., given amino acids in alignment positions) are present in sequence S and 0 otherwise. If the amino acids in community A are related to a given property in the protein family, then high values of Adh(S,A) indicate that sequence S may possess that property, being useful for gene annotation applications.

## Methods

### Alignment input

Multiple sequence alignments for protein families were obtained directly from PFAM [21]. In order to reduce the known phylogeny bias in correlation studies, alignments were culled according to an identity cutoff. If the chosen cutoff is 80%, every sequence was compared to all others and, every time two sequences had more than 80% identity, the smallest sequence was removed from the alignment. The alignments were also visually inspected to check for errors in the alignment process or the presence of small fragments. The Fe/Mn superoxide dismutase alignment presented 675 sequences using 80% as the identity cutoff, while the peroxidases alignment still presented 977 sequences using 70% as the identity cutoff. Since the C-type lysozymes/alpha-lactalbumins were chosen as a test case for the effects of low sampling, three alignments were used: one obtained after an 80% identity cutoff (162 sequences), a second after a 90% cutoff (256 sequences) and a third after a 95% cutoff (323 sequences).

### Correlation graph calculation

In order to be checked for correlation, given amino acids must be present in a significant number of sequences at both positions. This threshold was calculated as described in [10] and was found to be 30% for SODs, 20% for peroxidases and 30% for C-type lysozymes/alpha-lactalbumins. Given a pair (e.g., H15 and D58), the correlation score as described in the theory section was calculated for the two directions and then averaged. Each score and also the average must be above the user specified threshold in order to have that pair written into the output graph. A very high threshold may result in missing useful data, while a low threshold may include spurious results. A control procedure, described as supplemental material (Text S1), indicates that while the correlation score spread in real multiple sequence alignments present a smooth distribution, column shuffled alignments show a drastic fall in the maximum score observed. Therefore, no values higher than 5 (virtually all of them among 0 and 1) are expected as background for the protein families studied in this article. A threshold of 10 with Δf = 0.2 for SODs resulted in 26 pairs, while more restrictive criteria for peroxidases, with a threshold of 20 and Δf = 0.15 still resulted in 514 pairs. For C-type lysozymes/alpha-lactalbumins using Δf = 0.15 resulted in 3, 15 and 30 pairs (for the alignments obtained after identity cutoffs of 80%, 90% and 95%, respectively).

### Community detection

The software package Radatools [16], [19], [22] was used for community detection. We used ten repetitions of a taboo search followed by bootstrapping [19], Newman's fast algorithm for community detection [20] followed by another round of bootstrapping.

## Results

### Fe/Mn superoxide dismutases

**Figure 1:**
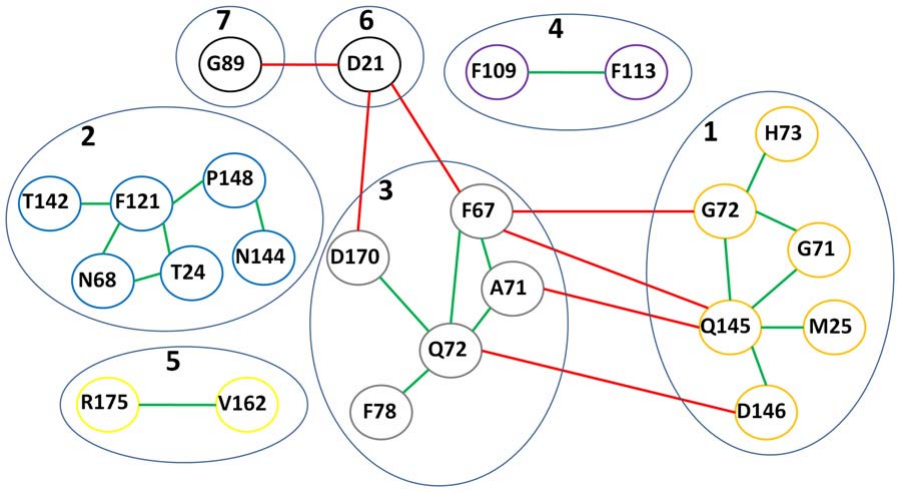
Network of correlations for the Fe-Mn superoxide dismutase family. The five groups (1–5) found after the community detection procedure have their members painted in orange, blue, gray, purple and yellow, respectively. The two isolated nodes or “one-member communities” (6 and 7) are painted in black. The edges represent correlation (green) or anti-correlation (red) above the threshold of 10 for the correlation score (see Methods).

Fe/Mn superoxide dismutases are an ideal test case for a methodology to detect and evaluate sets of residues defining functional characteristics in a protein family, since they simultaneously present mutually exclusive (as in iron versus manganese specificity) and independent (as in oligomeric state versus metal specificity) characteristics. After the procedures described in the methods section, a network of 23 vertices and 26 edges is generated, and after community analysis five groups and two individual residues arise as a total of seven communities. The resulting network is shown in figure 1.

The relation between the elements of a community can be represented in tabular form in *self-correlation matrices*, as shown in table 1, and by applying the ranking procedure to this community, we obtain an order of these residues, shown in table 2 (self-correlation matrices and ranking results for the other communities can be seen in the supplemental material, File S1, S2, 23, 24, S5, File S6, S7, S8, respectively). Finally, we can compute the values of Δ_AB_ to compare the different communities. The result is shown in table 3. The results in table 3 show that, from the five groups (communities 1–5), communities 1 and 3 are highly anti-correlated, while in all other cases they seem to present neither correlation nor anti-correlation, as demonstrated by values close to 0. The isolated residues 6 and 7 appear to be related, respectively, to communities 1 and 3, but were not placed in these communities because they lacked positive links to these two communities for the thresholds used.

**Table 2:** Member ranking for SOD community 1.

Element	Mean score
H73	18.2
D146	28.25
G72	40.83
M25	42.0
G71 Q145	59.0

H73 is the first to be eliminated, since its mean correlation score when compared to all other members is the lowest. After subsequent eliminations, the procedure ends when only G71 and Q145, which have a correlation score of 59, are present in the community.

**Table 3:** Δ_AB_ for the seven communities in Fe/Mn superoxide dismutases.

Community	1	2	3	4	5	6	7
1	26.08	−2.39	−32.8	−1.17	−0.75	11.83	−12.33
2	−2.78	15.31	6.7	2.92	11.08	5.17	1.33
3	−41.13	15.0	44.24	3.2	4.4	−30.0	22.2
4	−0.75	1.83	1.0	7.25	−0.5	0.5	0.0
5	−0.33	5.58	1.6	−0.5	12.5	3.0	0.0
6	23.17	13.33	−30.4	2.5	12.5	0.0	−41.0
7	−16.33	2.67	21.4	−0.5	0.0	−40.0	0.0

### Peroxidases-catalases

Peroxidases, like superoxide dismutases, are enzymes which are involved in oxidative stress, but have a large number of possible substrates – they simultaneously promote the oxidation of organic substrates while reducing H_2_O_2_, producing water. They are ubiquitous in living organisms and the latest release of PFAM contains 4028 peroxidase domain sequences distributed in 666 species. They are classically subdivided into three classes (I, II and III) based on phylogeny. Class I peroxidases are intracellular and present in prokaryotes, class II are extracellular and present in fungi, while class III are secretory and present in plants. Some other crucial features differentiate between them including the presence of calcium ions, disulfide bridges and glycosylation sites in class II and class III peroxidases. Being heme binding proteins, they present proximal and distal sites containing histidines, but varying in the other residues that constitute the tryads (usually, W/H/R and D/H/W for Class I peroxidases and F/H/R and D/H/F for classes II and III). These features, together with the previous observation of a high correlation among a large number of residues in the peroxidases family using the statistical coupling analysis metric [23] turns this family into an interesting case for the methodology described here. Even after limiting the alignment to sequences with at most 70% identity, calculation of correlated pairs with scores greater than 20 (or lower than −20, if anti-correlated) and Δf = 0.15 results in a graph with 514 edges between 113 vertices, a much larger network than the previous case. Network decomposition yielded six communities and 41 isolated residues. Some outliers (community members which are not highly correlated to other members) have been observed after network decomposition for some residues presenting only anti-correlations in the complete graph, turning this case into an interesting test for the subsequent methods as described above.

The first community is composed of 30 members, each one composed of residues ranging from 20% to 40% overall frequency and which, upon the presence of other members, increase their frequency to very high values, characterizing a well-formed community (Table 4).

**Table 4:** Self-correlation matrix for peroxidases.

POS	ALL	A49	A52	A152	D31	DX(483)	D233	F251	F119	F21	G252	G166	G176	H29	H40
A49	33.3	X	81.2	91.0	88.5	83.1	83.0	92.7	73.4	91.4	89.2	85.8	70.6	89.7	0.0
A52	21.2	51.7	X	80.1	77.0	58.2	70.3	80.0	46.8	81.2	77.5	76.3	39.3	77.9	0.0
A152	20.6	56.3	77.8	X	88.0	67.5	81.7	93.2	52.2	91.4	90.1	87.7	41.6	89.7	0.0
D31	22.2	59.1	80.7	95.0	X	69.5	83.0	95.1	54.0	98.0	92.0	89.1	43.0	96.2	1.0
DX(483)	25.5	63.7	70.0	83.6	79.7	X	74.2	83.9	64.2	82.7	80.8	80.6	68.2	80.8	0.0
D233	23.4	58.5	77.8	93.0	87.6	68.3	X	92.7	53.2	90.9	90.1	87.7	44.4	89.2	3.9
F251	21.0	58.5	79.2	95.0	89.9	69.1	83.0	X	53.2	93.4	93.0	90.0	43.9	91.5	0.0
F119	38.1	84.0	84.1	96.5	92.6	96.0	86.5	96.6	X	95.4	93.0	92.4	91.1	93.9	0.0
F21	20.2	55.4	77.3	89.6	88.9	65.5	78.2	89.8	50.5	X	86.9	83.9	39.7	90.1	2.0
G252	21.8	58.5	79.7	95.5	90.3	69.1	83.8	96.6	53.2	93.9	X	90.5	43.9	92.0	0.5
G166	21.6	55.7	77.8	92.0	86.6	68.3	80.8	92.7	52.4	89.8	89.7	X	43.0	88.3	1.0
G176	21.9	46.5	40.6	44.3	42.4	58.6	41.5	45.9	52.4	43.1	44.1	43.6	X	43.2	0.0
H29	21.8	58.8	80.2	95.0	94.5	69.1	83.0	95.1	53.8	97.5	92.0	89.1	43.0	X	0.0
H40	20.9	0.0	0.0	0.0	0.9	0.0	3.5	0.0	0.0	2.0	0.5	0.9	0.0	0.0	X

To facilitate visualization, only the first fourteen residues are shown. The full matrix is available as a supplemental material. Residue numbering corresponds to royal palm tree peroxidase. If a residue is not present in this protein, its numbering in the full alignment is shown within parentheses.

However, a clear outlier can be seen in the table: residue H40 is present in 20.9% of the sequences, but its rate drops to near-zero values upon the presence of other community members. By applying the ranking procedure (table 5), it is the first residue to be removed from the community, since its mean score when compared to other members is negative. All other members have positive mean values, and the last two residues have a positive score of 123 (due to the fact that the presence of the tryptophan increases the rate of the arginine from 22.3% to 97.2%, while the presence of the arginine increases the rate of the tryptophan from 21.9% to 95.4%).

**Table 5:** Member ranking for community 1 in peroxidases.

Element	Mean score
H40 (130)	−19.9
G176 (708)	14.0
F119 (469)	50.9
A49 (236)	52.1
D0 (483)	56.0
A52 (243)	65.6
I54 (246)	68.6
L155 (610)	81.1
T163 (624)	85.5
D233 (875)	87. 2
V76 (282)	88.0
V117 (465)	89.8
G166 (630)	96.9
W25 (93)	98.9
Q64 (262)	100.4
L61 (258)	104.5
T131 (481)	105.2
F21 (89)	107.2
A152 (604)	110.1
G252 (1018)	110.3
L257 (1033)	111.4
D31 (108)	113.3
R53 (244)	114.4
Q42 (133)	115.2
L28 (96)	115.3
H29 (106)	115.5
PX (137)	116.3
F251 (1017)	122.5
R169 (698) WX (887)	123.0

Numbering corresponds to royal palm tree peroxidase and alignment numbering is given between parentheses.

The second community also presents three outliers, which are also readily eliminated by the ranking procedure. The calculation of Δ_AB_ for peroxidases is shown in table 6.

**Table 6:** Δ_AB_ matrix for peroxidase communities.

Community	1	2	3	4	5	6
1	71.2	−24.4	−66.9	56.7	65.7	−11.9
2	−21.9	34.4	22.2	−41.5	−40.3	−15.1
3	−42.5	9.0	39.9	−11.7	−17.9	5.6
4	35.9	−43.6	−19.6	68.2	77.6	17.1
5	49.7	−44.0	−30.8	100.9	79.0	6.0
6	−12.0	−17.7	19.4	25.2	7.2	50.5

This matrix refers to communities having at least two members. The full matrix is available as supplemental material.

### C-type lysozymes/alpha-lactalbumins

The family of C-type lysozymes/alpha-lactalbumins have an interesting evolutionary history. Being a potent agent against bacteria, an ancestral lysozyme gene suffered a duplication about 300–400 million years ago, which resulted in a new protein that codes for alpha-lactalbumin. This proteins lacks the lysozyme catalytic activity, but can associate with β-1,4-galactosyltransferase, forming a functional heterodimer known as *lactose synthase*. Furthermore, some members of the family show calcium binding capacity while others do not – a property which is not truly independent of the presence of lysozyme activity since lactalbumins seem to have originated from calcium binding proteins after an early separation of calcium binding/non calcium binding members [24].

**Figure 2:**
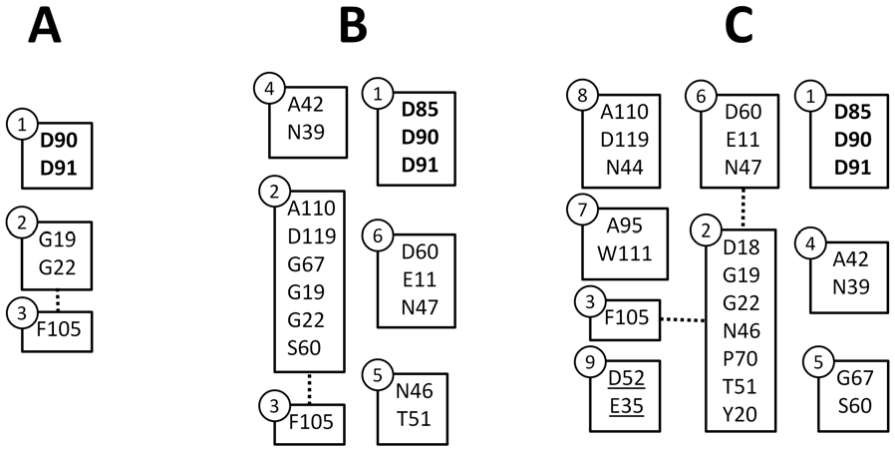
Community structure for the networks calculated for C-type lysozymes/alpha-lactalbumins. Data was calculated using as identity cutoff A) 80% B) 90% and C) 95%. Residues shown in the same box were grouped into a single community. If, for two communities A and B, both ΔAB, ΔBA and (ΔAB+ΔBA)/2 are higher than the chosen pair-wise correlation score cutoff (in this case, 10), the two boxes for communities A and B are connected by a dotted line if (ΔAB+ΔBA)/2 is negative (no positive values were found). Residues involved in calcium binding are shown in bold, and residues involved in lysozyme activity are underlined (see *Discussion*).

The networks for this family are much smaller than those described for the two previous examples. Using the alignment obtained after an 80% identity cutoff, there are only five vertices and three edges. For a 90% identity cutoff, there are 17 vertices and 15 edges, and, finally, a network obtained from an alignment after a 95% cutoff consisted of 25 vertices and 30 edges. It should be noted that the number of edges is relatively low given the number of vertices, which may be explained by the fact that the number of sequences used is much smaller, implying fewer correlated pairs falling within the criteria used. The decomposition of these small networks into communities is shown in figure 2.

Even though low sampling is clearly a problem, it was still possible to obtain useful information from these data, as will be described below.

## Discussion

### Structural and functional interpretation of correlation results

**Figure 3:**
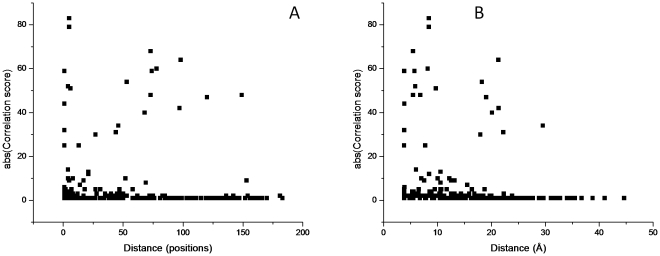
Effect of pair-wise distance on correlation. A) linear distance vs. absolute correlation B) three-dimensional distance vs. absolute correlation.

**Figure 4:**
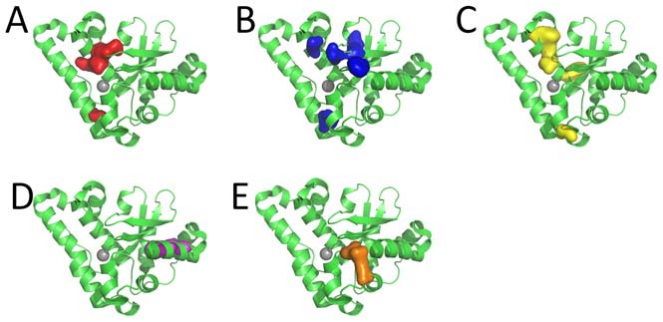
Correlated residue communities in Fe/Mn Superoxide dismutases. A–E refer to communities 1–5, respectively. The metal ion is shown as a grey sphere. PDB code: 3ESF.

The detection of contact pairs was the first application for correlated mutation detection [1]. However, even though it is common to see high correlation between residues in contact, many times high correlation is observed between residues which are not close. Correlation vs. distance plots for Fe/Mn superoxide dismutases can be seen in Figure 3 (linear distance in 3A, three-dimensional distance in 3B). Even though some of the highest correlated pairs are either close in the sequence or in the 3D structure, highly correlated pairs can still be seen for pairs which are considerably distant in both cases. A linear fit (distance = a–b*correlation) and an ANOVA analysis on both datasets showed that there is an observed statistical significance for three-dimensional distances (a = 13.6±4 and b = −0.4±0.2), but the value for the linear coefficient b is too small for any practical use. No statistical significance was observed when linear distances were used. Curiously, if communities are analyzed separately, it can be seen that the two communities having only two residues each (4 and 5) in fact report contact pairs, while this is not the case for communities 1–3, as seen in figure 4.

The observed pattern for Fe/Mn superoxide dismutases can be readily interpreted, however, when analyzed at the light of the well-defined characteristics in this protein family. In figure 1, it is possible to immediately identify the functional connection to the network decomposition into communities. Community 1 groups six residues which are related to the presence of an active-site manganese, while community 3 groups residues found in SODs which bind iron instead. Since these properties are mostly mutually exclusive (except for the rare cambialistic SODs), the residues in their communities are linked with negative edges, shown in red. As previously noted, an overall correlation measure would miss that feature, since positions 71 and 72 appear in *both* communities, but with different residue types. Community 2 groups six residues which are related to dimeric SODs. It should be noted that they do not present either positive or negative links to the other communities, which is compatible with the notion that metal selectivity and oligomeric state are independent properties. Table 3, containing Δ_AB_ values, quantifies the scenario which could readily identified simply by examining figure 1: the fact that the values for Δ_13_ and Δ_31_ are high and negative is expected, since communities 1 and 3 are related to mutually exclusive characteristics (the binding of iron or manganese). On the other hand, the values for Δ_AB_ relating community 2 to communities 1 and 3 are for most cases much smaller, consistent with the fact that the oligomeric state property should be unrelated to metal specificity.

**Figure 5:**
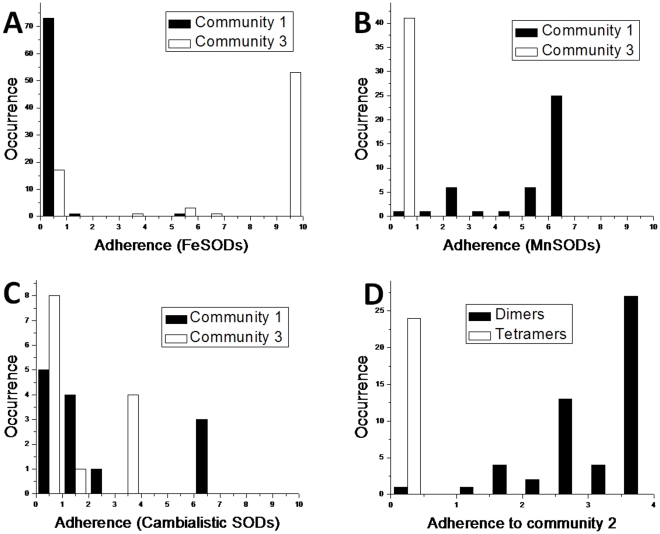
Adherence value histograms for Fe/Mn superoxide dismutases. A) FeSODs, communities 1 and 3; B) MnSODs, communities 1 and 3; C) Cambialistic SODs, communities 1 and 3; D) Dimeric and tetrameric SODs, community 2.

Since the Fe/Mn superoxide dismutase family presents a good test set for oligomeric state and metal specificity [12], we can also assess the utility of the adherence parameter Adh(S,A) as defined above. Adh(S,A) was calculated for every sequence in the set, and the results are shown as histograms in Figure 5. Each one refers to sequences with a given characteristic – dimeric, tetrameric, iron-binding and manganese binding.

For iron binding SODs, virtually all sequences have Adh(S,1) equal to zero and the maximum value for Adh(S,3) (Figure 5A). Conversely, all manganese binding SODs have Adh(S,3) equal to zero, and most have high values for Adh(S,1). Most cambialistic SODs have low or null values for Adh(S,1) and Adh(S,3), suggesting that the lack of residues in communities 1 and 3 are related to non-specificity for manganese or iron. Finally, all tetrameric SODs have Adh(S,2) equal to zero, while most dimeric SODs show higher values.

The six communities observed for peroxidases-catalases show residues whose separation can be readily interpreted. Community 2 groups typical residues from a Class III peroxidase: the most striking ones are the six cysteines involved in disulfide bridges in this class. It also presents F41, a phenylalanine on the distal side of the heme which hinders access to the heme iron (a typical feature of class III peroxidases). Community 3 groups F152, H42, H169 and R38. These residues are present in most peroxidase binding sites: R38 and H42 make direct contacts with the peroxide and F152 contacts the heme group, while H169 is the heme proximal histidine. However, since not all peroxidases have the same catalityc triad, the differences can also be seen in the community structure: contrary to class III peroxidases, class I members present a tryptophan in position 41 instead of a phenylalanine, and, therefore, while F41 is present in community 2, W41 forms community 4 with three other residues (E110, P63 and R55). Class I peroxidases tend to have a different organization. They are homodimers in which each chain is composed of two peroxidase domains in tandem. From the three other residues in community 4, the proline and arginine are in the dimer interface, while the glutamic acid is part of a salt bridge network connecting the two peroxidase domains of a single chain, as seen in the crystal structure of *H. marismortui* catalase-peroxidase (PDB code: 1ITK). The C-terminal domain of class I peroxidases also present specifically conserved residues, which are grouped under community 1. Since the exact function of this domain is not perfectly understood, the roles of the residues in this community cannot be readily interpreted. However, it has been shown that, albeit inactive, the second domain is crucial to the dual peroxidase-catalase activity of Class I peroxidases [25], and therefore some evolutionary pressure on key residues must still be present in the C-terminal domain. This analysis, therefore, suggests positions which could be further investigated in order to understand the function of this domain. Finally, community 6 is formed by an arginine and a glutamic acid forming a salt bridge in KATGs, a catalase/peroxidase subfamily found among Class I members, and the residues in community 5 are the charged pair D105 and R107 and a tryptophan residue, W123, also present in most KATGs – the first two residues do not have structural equivalents in class II and class III peroxidases, since they are in an inserted loop. The relation between all those classes are consistent with the Δ_AB_ values found in table 6. The complete self-correlation matrices and member rankings for all communities discussed above is provided as supplementary material (files S9, S10, S11, S12, S13, S14, S15, S16, S17, S18, S19).

The case of C-type lysozymes/alpha-lactalbumins is a nice example to illustrate the effect of poor sampling in our proposed methodology. There are two properties which are well characterized in this family and should, in principle arise in our analysis assuming the validity of our hypothesis – that the presence of different characteristics in a protein family will result in the formation of communities grouping the residues involved in those characteristics. The first property is lysozyme activity, which is lost in alpha-lactalbumins, and the second is the ability to bind calcium. In figure 2, we see how the formation of correlated pairs and communities evolves when using different identity cutoffs. When using 80%, the same value used for Fe/Mn-SODs, there are very few residues passing the criteria, but it is already possible to identify a pair whose function can be readily interpreted: D90 and D91 are involved in calcium binding. When less stringent identity cutoffs are used, D85 (which also coordinates the calcium ion) joins this community, and the lysozyme catalytic pair D52-E35 arises. However, this is at the expense of the appearance of other residue communities whose roles cannot yet be readily interpreted. In the search for novel communities it is therefore probably prudent to employ more than one identity cutoff and examine its effect on the results if the number of sequences is limited.

The utilization of a residue-specific correlation metric followed by community analysis can capture sub-class determinant features that will not be observed using traditional methods described earlier. Since they usually report only the overall dependence of two columns in an alignment, all the amino acid specific information will be lost. In order to exemplify this, we generated correlation data with three different methods: McBASC (the original correlation metric proposed by Göbel et al. [1]), Mutual Information (the leading method used nowadays for correlation studies, usually to find contact pairs) and ELSC, a perturbation-based method which uses explicit likelihood to calculate correlations [8]. Since all these methods will report a correlation value for each pair of positions, we have to apply a cutoff in order to generate networks. Two cutoffs (50% and 80% of the maximum correlation value found) were used for each method, and the results (Text S2) show that only McBasc report communities related to metal binding and oligomeric state, and the metal-specific information is lost (since the metric is not residue-specific, there is only a single community for metal related positions). These comparisons highlight the advantages of the method we describe here.

### Conclusions

We present a method based on correlated mutations and network analysis to calculate and analyze groups of amino acids which may be related to functional classes in protein families. Due to nature of the correlation metric and the network decomposition method, the results can be readily interpreted and related to biological features and their inter-relation (independence/mutual exclusivity). We also propose additional parameters and procedures that can be used to further analyze and extract information from the data. We argue that community structure in networks constructed using the described method is an expected feature for protein families presenting functional sub-classes, and therefore could be exploited to identify key residues for specific functional properties. Also, it can be a useful tool for gene annotation, since key residues which are clustered in a community should be more likely to predict function than sequence identity methods, which considers all residues evenly. The programs used for the presented method are available to academic users upon request.

## Supporting Information

File S1 **Self-correlation matrix for SODs community 1.**
(HTML)

File S2 **Self-correlation matrix for SODs community 2.**
(HTML)

File S3 **Self-correlation matrix for SODs community 3.**
(HTML)

File S4 **Self-correlation matrix for SODs community 4.**
(HTML)

File S5 **Self-correlation matrix for SODs community 5.**
(HTML)

File S6 **Member ranking for SODs community 1.**
(HTML)

File S7 **Member ranking for SODs community 2.**
(HTML)

File S8 **Member ranking for SODs community 3.**
(HTML)

File S9 **Self-correlation matrix for Peroxidases community 1.**
(HTML)

File S10 **Self-correlation matrix for Peroxidases community 2.**
(HTML)

File S11 **Self-correlation matrix for Peroxidases community 3.**
(HTML)

File S12 **Self-correlation matrix for Peroxidases community 4.**
(HTML)

File S13 **Self-correlation matrix for Peroxidases community 5.**
(HTML)

File S14 **Self-correlation matrix for Peroxidases community 6.**
(HTML)

File S15 **Member ranking for Peroxidases community 1.**
(HTML)

File S16 **Member ranking for Peroxidases community 2.**
(HTML)

File S17 **Member ranking for Peroxidases community 3.**
(HTML)

File S18 **Member ranking for Peroxidases community 4.**
(HTML)

File S19 **Member ranking for Peroxidases community 5.**
(HTML)

Text S1 **Figure 1: Distribution of correlation scores for the Fe/Mn-SODs protein family.** Figure 2: Correlation score spread (in logarithmic scale) for Fe/Mn-SODs (black) and 1000 alignments generated by shuffling the columns from the Fe/Mn-SODs alignment (red).Figure 3: Correlation score spread for 1000 shuffled alignments for (A) Peroxidases-catalases and (B) C-type lysozymes/lactalbumins.
(DOC)

Text S2 **Table 1: Network decomposition for McBASC, cutoff = 0.5maximum.** Table 2: Network decomposition for McBASC, cutoff = 0.8maximum. Table 3: Network decomposition for mutual information, cutoff = 0.8maximum. Table 4: Network decomposition for ELSC, cutoff = 0.8maximum.
(DOC)
