# Supplementary material for: Using Amino Acid Correlation and Community Detection Algorithms to Identify Functional Determinants in Protein Families
Source: PLoS One. 2011 Dec 20;6(12):e27786. doi: 10.1371/journal.pone.0027786 (PMC3243672; doi:10.1371/journal.pone.0027786)
Supplement: File S1 — Self-correlation matrix for SODs community 1. (HTML) [file pone.0027786.s001.html]

| POS | ALL | D146 | G71 | G72 | H73 | M25 | Q145 |
| --- | --- | --- | --- | --- | --- | --- | --- |
| **D146** | 54.1 | X | 78.4 | 73.1 | 66.7 | 81.7 | 89.7 |||  |  |  |  |  |  |  |  |  |  |  |  |  |  |  |  |  |  |  |  |  |  |  |  |  |  |  |  |  |  |  |  |  |  |  |  |  |  |  |  |
| --- | --- | --- | --- | --- | --- | --- | --- | --- | --- | --- | --- | --- | --- | --- | --- | --- | --- | --- | --- | --- | --- | --- | --- | --- | --- | --- | --- | --- | --- | --- | --- | --- | --- | --- | --- | --- | --- | --- | --- |
| **G71** | 60.4 | 87.7 | X | 86.1 | 81.4 | 93.3 | 99.7 |||  |  |  |  |  |  |  |  |  |  |  |  |  |  |  |  |  |  |  |  |  |  |  |  |  |  |  |  |  |  |  |  |
| --- | --- | --- | --- | --- | --- | --- | --- | --- | --- | --- | --- | --- | --- | --- | --- | --- | --- | --- | --- | --- | --- | --- | --- | --- | --- | --- | --- | --- | --- | --- | --- |
| **G72** | 63.9 | 86.3 | 90.9 | X | 89.1 | 97.7 | 99.4 |||  |  |  |  |  |  |  |  |  |  |  |  |  |  |  |  |  |  |  |  |  |  |  |  |
| --- | --- | --- | --- | --- | --- | --- | --- | --- | --- | --- | --- | --- | --- | --- | --- | --- | --- | --- | --- | --- | --- | --- | --- |
| **H73** | 57.3 | 70.7 | 77.2 | 80.0 | X | 85.8 | 79.4 |||  |  |  |  |  |  |  |  |  |  |  |  |  |  |  |  |
| --- | --- | --- | --- | --- | --- | --- | --- | --- | --- | --- | --- | --- | --- | --- | --- |
| **M25** | 51.1 | 77.3 | 78.9 | 78.2 | 76.5 | X | 87.7 |||  |  |  |  |  |  |  |  |
| --- | --- | --- | --- | --- | --- | --- | --- |
| **Q145** | 51.7 | 85.8 | 85.3 | 80.5 | 71.6 | 88.7 | X ||
