# Supplementary material for: Using Amino Acid Correlation and Community Detection Algorithms to Identify Functional Determinants in Protein Families
Source: PLoS One. 2011 Dec 20;6(12):e27786. doi: 10.1371/journal.pone.0027786 (PMC3243672; doi:10.1371/journal.pone.0027786)
Supplement: File S2 — Self-correlation matrix for SODs community 2. (HTML) [file pone.0027786.s002.html]

| POS | ALL | F121 | N144 | N68 | P148 | T142 | T24 |
| --- | --- | --- | --- | --- | --- | --- | --- |
| **F121** | 63.9 | X | 74.9 | 99.2 | 85.4 | 80.3 | 92.9 |||  |  |  |  |  |  |  |  |  |  |  |  |  |  |  |  |  |  |  |  |  |  |  |  |  |  |  |  |  |  |  |  |  |  |  |  |  |  |  |  |
| --- | --- | --- | --- | --- | --- | --- | --- | --- | --- | --- | --- | --- | --- | --- | --- | --- | --- | --- | --- | --- | --- | --- | --- | --- | --- | --- | --- | --- | --- | --- | --- | --- | --- | --- | --- | --- | --- | --- | --- |
| **N144** | 73.8 | 86.5 | X | 85.6 | 87.1 | 85.4 | 84.0 |||  |  |  |  |  |  |  |  |  |  |  |  |  |  |  |  |  |  |  |  |  |  |  |  |  |  |  |  |  |  |  |  |
| --- | --- | --- | --- | --- | --- | --- | --- | --- | --- | --- | --- | --- | --- | --- | --- | --- | --- | --- | --- | --- | --- | --- | --- | --- | --- | --- | --- | --- | --- | --- | --- |
| **N68** | 55.6 | 86.3 | 64.5 | X | 74.9 | 68.8 | 80.9 |||  |  |  |  |  |  |  |  |  |  |  |  |  |  |  |  |  |  |  |  |  |  |  |  |
| --- | --- | --- | --- | --- | --- | --- | --- | --- | --- | --- | --- | --- | --- | --- | --- | --- | --- | --- | --- | --- | --- | --- | --- |
| **P148** | 71.0 | 94.9 | 83.7 | 95.7 | X | 84.0 | 89.4 |||  |  |  |  |  |  |  |  |  |  |  |  |  |  |  |  |
| --- | --- | --- | --- | --- | --- | --- | --- | --- | --- | --- | --- | --- | --- | --- | --- |
| **T142** | 64.0 | 80.5 | 74.1 | 79.2 | 75.8 | X | 78.5 |||  |  |  |  |  |  |  |  |
| --- | --- | --- | --- | --- | --- | --- | --- |
| **T24** | 62.8 | 91.4 | 71.5 | 91.5 | 79.1 | 77.1 | X ||
