# Supplementary material for: Using Amino Acid Correlation and Community Detection Algorithms to Identify Functional Determinants in Protein Families
Source: PLoS One. 2011 Dec 20;6(12):e27786. doi: 10.1371/journal.pone.0027786 (PMC3243672; doi:10.1371/journal.pone.0027786)
Supplement: File S3 — Self-correlation matrix for SODs community 3. (HTML) [file pone.0027786.s003.html]

| POS | ALL | A71 | D170 | F67 | F78 | Q72 |
| --- | --- | --- | --- | --- | --- | --- |
| **A71** | 30.2 | X | 67.7 | 81.7 | 76.6 | 80.5 |||  |  |  |  |  |  |  |  |  |  |  |  |  |  |  |  |  |  |  |  |  |  |  |  |  |  |  |  |
| --- | --- | --- | --- | --- | --- | --- | --- | --- | --- | --- | --- | --- | --- | --- | --- | --- | --- | --- | --- | --- | --- | --- | --- | --- | --- | --- | --- |
| **D170** | 41.3 | 92.6 | X | 99.0 | 84.1 | 97.0 |||  |  |  |  |  |  |  |  |  |  |  |  |  |  |  |  |  |  |  |  |  |
| --- | --- | --- | --- | --- | --- | --- | --- | --- | --- | --- | --- | --- | --- | --- | --- | --- | --- | --- | --- | --- |
| **F67** | 30.8 | 83.3 | 73.8 | X | 74.5 | 89.2 |||  |  |  |  |  |  |  |  |  |  |  |  |  |  |
| --- | --- | --- | --- | --- | --- | --- | --- | --- | --- | --- | --- | --- | --- |
| **F78** | 35.4 | 89.7 | 72.0 | 85.6 | X | 84.4 |||  |  |  |  |  |  |  |
| --- | --- | --- | --- | --- | --- | --- |
| **Q72** | 34.2 | 91.2 | 80.3 | 99.0 | 81.6 | X ||
