# Supplementary material for: Using Amino Acid Correlation and Community Detection Algorithms to Identify Functional Determinants in Protein Families
Source: PLoS One. 2011 Dec 20;6(12):e27786. doi: 10.1371/journal.pone.0027786 (PMC3243672; doi:10.1371/journal.pone.0027786)
Supplement: File S4 — Self-correlation matrix for SODs community 4. (HTML) [file pone.0027786.s004.html]

| POS | ALL | F109 | F113 |
| --- | --- | --- | --- |
| **F109** | 74.8 | X | 88.7 |||  |  |  |  |
| --- | --- | --- | --- |
| **F113** | 75.9 | 89.9 | X ||
