# Supplementary material for: Using Amino Acid Correlation and Community Detection Algorithms to Identify Functional Determinants in Protein Families
Source: PLoS One. 2011 Dec 20;6(12):e27786. doi: 10.1371/journal.pone.0027786 (PMC3243672; doi:10.1371/journal.pone.0027786)
Supplement: File S5 — Self-correlation matrix for SODs community 5. (HTML) [file pone.0027786.s005.html]

| POS | ALL | R175 | V162 |
| --- | --- | --- | --- |
| **R175** | 81.5 | X | 98.0 |||  |  |  |  |
| --- | --- | --- | --- |
| **V162** | 75.7 | 91.1 | X ||
