# Supplementary material for: Using Amino Acid Correlation and Community Detection Algorithms to Identify Functional Determinants in Protein Families
Source: PLoS One. 2011 Dec 20;6(12):e27786. doi: 10.1371/journal.pone.0027786 (PMC3243672; doi:10.1371/journal.pone.0027786)
Supplement: File S6 — Member ranking for SODs community 1. (HTML) [file pone.0027786.s006.html]

|  |  |  |  |  |  |  |  |  |  |  |  |
| --- | --- | --- | --- | --- | --- | --- | --- | --- | --- | --- | --- |
| **Element** | Mean score || **H73 (841)** | 18.200001 |
| **D146 (1242)** | 28.250000 |
| **G72 (840)** | 40.833332 |
| **M25 (701)** | 42.000000 |
| **G71 (833) Q145 (1241)** | 59.000000 |
