# Supplementary material for: Using Amino Acid Correlation and Community Detection Algorithms to Identify Functional Determinants in Protein Families
Source: PLoS One. 2011 Dec 20;6(12):e27786. doi: 10.1371/journal.pone.0027786 (PMC3243672; doi:10.1371/journal.pone.0027786)
Supplement: File S7 — Member ranking for SODs community 2. (HTML) [file pone.0027786.s007.html]

|  |  |  |  |  |  |  |  |  |  |  |  |
| --- | --- | --- | --- | --- | --- | --- | --- | --- | --- | --- | --- |
| **Element** | Mean score || **N144 (1240)** | 7.300000 |
| **T142 (1089)** | 9.625000 |
| **P148 (1244)** | 24.166666 |
| **T24 (700)** | 37.000000 |
| **F121 (927) N68 (829)** | 54.000000 |
