# Supplementary material for: Using Amino Acid Correlation and Community Detection Algorithms to Identify Functional Determinants in Protein Families
Source: PLoS One. 2011 Dec 20;6(12):e27786. doi: 10.1371/journal.pone.0027786 (PMC3243672; doi:10.1371/journal.pone.0027786)
Supplement: File S8 — Member ranking for SODs community 3. (HTML) [file pone.0027786.s008.html]

|  |  |  |  |  |  |  |  |  |  |
| --- | --- | --- | --- | --- | --- | --- | --- | --- | --- |
| **Element** | Mean score || **F78 (848)** | 46.750000 |
| **A71 (833)** | 52.333332 |
| **D170 (1431)** | 63.000000 |
| **F67 (828) Q72 (840)** | 83.000000 |
