# Supplementary material for: Using Amino Acid Correlation and Community Detection Algorithms to Identify Functional Determinants in Protein Families
Source: PLoS One. 2011 Dec 20;6(12):e27786. doi: 10.1371/journal.pone.0027786 (PMC3243672; doi:10.1371/journal.pone.0027786)
Supplement: File S11 — Self-correlation matrix for Peroxidases community 3. (HTML) [file pone.0027786.s011.html]

| POS | ALL | F152 | H42 | H169 | R38 |
| --- | --- | --- | --- | --- | --- |
| **F152** | 69.4 | X | 92.6 | 92.4 | 92.0 |||  |  |  |  |  |  |  |  |  |  |  |  |  |  |  |  |  |  |
| --- | --- | --- | --- | --- | --- | --- | --- | --- | --- | --- | --- | --- | --- | --- | --- | --- | --- |
| **H42** | 64.6 | 86.1 | X | 86.5 | 97.3 |||  |  |  |  |  |  |  |  |  |  |  |  |
| --- | --- | --- | --- | --- | --- | --- | --- | --- | --- | --- | --- |
| **H169** | 72.6 | 96.6 | 97.1 | X | 95.9 |||  |  |  |  |  |  |
| --- | --- | --- | --- | --- | --- |
| **R38** | 65.0 | 86.1 | 97.9 | 85.9 | X ||
