# Supplementary material for: Using Amino Acid Correlation and Community Detection Algorithms to Identify Functional Determinants in Protein Families
Source: PLoS One. 2011 Dec 20;6(12):e27786. doi: 10.1371/journal.pone.0027786 (PMC3243672; doi:10.1371/journal.pone.0027786)
Supplement: File S12 — Self-correlation matrix for Peroxidases community 4. (HTML) [file pone.0027786.s012.html]

| POS | ALL | E110 | P63 | R55 | W41 |
| --- | --- | --- | --- | --- | --- |
| **E110** | 39.4 | X | 87.5 | 89.7 | 91.0 |||  |  |  |  |  |  |  |  |  |  |  |  |  |  |  |  |  |  |
| --- | --- | --- | --- | --- | --- | --- | --- | --- | --- | --- | --- | --- | --- | --- | --- | --- | --- |
| **P63** | 37.6 | 83.4 | X | 87.9 | 90.5 |||  |  |  |  |  |  |  |  |  |  |  |  |
| --- | --- | --- | --- | --- | --- | --- | --- | --- | --- | --- | --- |
| **R55** | 35.6 | 81.0 | 83.4 | X | 88.4 |||  |  |  |  |  |  |
| --- | --- | --- | --- | --- | --- |
| **W41** | 38.7 | 89.4 | 93.2 | 96.0 | X ||
