# Supplementary material for: Using Amino Acid Correlation and Community Detection Algorithms to Identify Functional Determinants in Protein Families
Source: PLoS One. 2011 Dec 20;6(12):e27786. doi: 10.1371/journal.pone.0027786 (PMC3243672; doi:10.1371/journal.pone.0027786)
Supplement: File S13 — Self-correlation matrix for Peroxidases community 5. (HTML) [file pone.0027786.s013.html]

| POS | ALL | DX(231) | RX(233) | W68 |
| --- | --- | --- | --- | --- |
| **DX(231)** | 31.8 | X | 95.1 | 93.6 |||  |  |  |  |  |  |  |  |  |  |
| --- | --- | --- | --- | --- | --- | --- | --- | --- | --- |
| **RX(233)** | 31.1 | 92.9 | X | 92.7 |||  |  |  |  |  |
| --- | --- | --- | --- | --- |
| **W68** | 32.0 | 94.2 | 95.4 | X ||
