# Supplementary material for: Using Amino Acid Correlation and Community Detection Algorithms to Identify Functional Determinants in Protein Families
Source: PLoS One. 2011 Dec 20;6(12):e27786. doi: 10.1371/journal.pone.0027786 (PMC3243672; doi:10.1371/journal.pone.0027786)
Supplement: File S14 — Self-correlation matrix for Peroxidases community 6. (HTML) [file pone.0027786.s014.html]

| POS | ALL | D159 | R149 |
| --- | --- | --- | --- |
| **D159** | 20.7 | X | 91.5 |||  |  |  |  |
| --- | --- | --- | --- |
| **R149** | 20.4 | 90.1 | X ||
