# Supplementary material for: Using Amino Acid Correlation and Community Detection Algorithms to Identify Functional Determinants in Protein Families
Source: PLoS One. 2011 Dec 20;6(12):e27786. doi: 10.1371/journal.pone.0027786 (PMC3243672; doi:10.1371/journal.pone.0027786)
Supplement: File S15 — Member ranking for Peroxidases community 1. (HTML) [file pone.0027786.s015.html]

|  |  |  |  |  |  |  |  |  |  |  |  |  |  |  |  |  |  |  |  |  |  |  |  |  |  |  |  |  |  |  |  |  |  |  |  |  |  |  |  |  |  |  |  |  |  |  |  |  |  |  |  |  |  |  |  |  |  |  |  |
| --- | --- | --- | --- | --- | --- | --- | --- | --- | --- | --- | --- | --- | --- | --- | --- | --- | --- | --- | --- | --- | --- | --- | --- | --- | --- | --- | --- | --- | --- | --- | --- | --- | --- | --- | --- | --- | --- | --- | --- | --- | --- | --- | --- | --- | --- | --- | --- | --- | --- | --- | --- | --- | --- | --- | --- | --- | --- | --- | --- |
| **Element** | Mean score || **H40 (130)** | -19.931034 |
| **G176 (708)** | 14.035714 |
| **F119 (469)** | 50.944443 |
| **A49 (236)** | 52.153847 |
| **D0 (483)** | 56.040001 |
| **A52 (243)** | 65.604164 |
| **I54 (246)** | 68.630432 |
| **L155 (610)** | 81.090912 |
| **T163 (624)** | 85.500000 |
| **D233 (875)** | 87.150002 |
| **V76 (282)** | 88.052635 |
| **V117 (465)** | 89.777779 |
| **G166 (630)** | 96.911766 |
| **W25 (93)** | 98.906250 |
| **Q64 (262)** | 100.400002 |
| **L61 (258)** | 104.464287 |
| **T131 (481)** | 105.192307 |
| **F21 (89)** | 107.166664 |
| **A152 (604)** | 110.090912 |
| **G252 (1018)** | 110.300003 |
| **L257 (1033)** | 111.388885 |
| **D31 (108)** | 113.312500 |
| **R53 (244)** | 114.428574 |
| **Q42 (133)** | 115.250000 |
| **L28 (96)** | 115.300003 |
| **H29 (106)** | 115.500000 |
| **P0 (137)** | 116.333336 |
| **F251 (1017)** | 122.500000 |
| **R169 (698) W0 (887)** | 123.000000 |
