# Supplementary material for: Using Amino Acid Correlation and Community Detection Algorithms to Identify Functional Determinants in Protein Families
Source: PLoS One. 2011 Dec 20;6(12):e27786. doi: 10.1371/journal.pone.0027786 (PMC3243672; doi:10.1371/journal.pone.0027786)
Supplement: File S16 — Member ranking for Peroxidases community 2. (HTML) [file pone.0027786.s016.html]

|  |  |  |  |  |  |  |  |  |  |  |  |  |  |  |  |  |  |  |  |  |  |  |  |  |  |  |  |  |  |  |  |  |  |  |  |  |  |  |  |  |  |  |  |  |  |  |  |  |  |  |  |  |  |  |  |  |  |
| --- | --- | --- | --- | --- | --- | --- | --- | --- | --- | --- | --- | --- | --- | --- | --- | --- | --- | --- | --- | --- | --- | --- | --- | --- | --- | --- | --- | --- | --- | --- | --- | --- | --- | --- | --- | --- | --- | --- | --- | --- | --- | --- | --- | --- | --- | --- | --- | --- | --- | --- | --- | --- | --- | --- | --- | --- | --- |
| **Element** | Mean score || **L249 (1015)** | -40.696430 |
| **F87 (319)** | -32.111111 |
| **V107 (382)** | -24.769230 |
| **P212 (772)** | 31.320000 |
| **W117 (465)** | 31.916666 |
| **N188 (722)** | 32.413044 |
| **Q249 (1015)** | 35.545456 |
| **D56 (248)** | 38.690475 |
| **G126 (476)** | 42.200001 |
| **L244 (1008)** | 44.000000 |
| **E64 (262)** | 46.527779 |
| **A68 (266)** | 51.382355 |
| **F77 (283)** | 58.437500 |
| **S52 (243)** | 59.133335 |
| **F41 (132)** | 61.071430 |
| **D50 (237)** | 62.384617 |
| **P92 (333)** | 66.291664 |
| **L54 (246)** | 69.363640 |
| **L204 (754)** | 72.050003 |
| **R182 (716)** | 78.666664 |
| **C208 (768)** | 81.812500 |
| **E88 (320)** | 85.142860 |
| **C44 (185)** | 91.250000 |
| **C176 (708)** | 96.099998 |
| **D107 (382)** | 100.750000 |
| **R106 (381)** | 101.166664 |
| **C49 (236)** | 115.000000 |
| **C91 (330) C97 (346)** | 135.500000 |
