# Supplementary material for: Using Amino Acid Correlation and Community Detection Algorithms to Identify Functional Determinants in Protein Families
Source: PLoS One. 2011 Dec 20;6(12):e27786. doi: 10.1371/journal.pone.0027786 (PMC3243672; doi:10.1371/journal.pone.0027786)
Supplement: File S17 — Member ranking for Peroxidases community 3. (HTML) [file pone.0027786.s017.html]

|  |  |  |  |  |  |  |  |
| --- | --- | --- | --- | --- | --- | --- | --- |
| **Element** | Mean score || **F152 (604)** | 44.333332 |
| **H169 (698)** | 46.750000 |
| **H42 (133) R38 (128)** | 92.500000 |
