# Supplementary material for: Using Amino Acid Correlation and Community Detection Algorithms to Identify Functional Determinants in Protein Families
Source: PLoS One. 2011 Dec 20;6(12):e27786. doi: 10.1371/journal.pone.0027786 (PMC3243672; doi:10.1371/journal.pone.0027786)
Supplement: File S18 — Member ranking for Peroxidases community 4. (HTML) [file pone.0027786.s018.html]

|  |  |  |  |  |  |  |  |
| --- | --- | --- | --- | --- | --- | --- | --- |
| **Element** | Mean score || **E110 (385)** | 84.000000 |
| **P63 (260)** | 92.750000 |
| **R55 (247) W41 (132)** | 108.000000 |
