# Supplementary material for: Using Amino Acid Correlation and Community Detection Algorithms to Identify Functional Determinants in Protein Families
Source: PLoS One. 2011 Dec 20;6(12):e27786. doi: 10.1371/journal.pone.0027786 (PMC3243672; doi:10.1371/journal.pone.0027786)
Supplement: File S19 — Member ranking for Peroxidases community 5. (HTML) [file pone.0027786.s019.html]

|  |  |  |  |  |  |
| --- | --- | --- | --- | --- | --- |
| **Element** | Mean score || **D0 (231)** | 118.250000 |
| **R0 (233) W68 (266)** | 119.000000 |
