# Supplementary material for: Using Amino Acid Correlation and Community Detection Algorithms to Identify Functional Determinants in Protein Families
Source: PLoS One. 2011 Dec 20;6(12):e27786. doi: 10.1371/journal.pone.0027786 (PMC3243672; doi:10.1371/journal.pone.0027786)
Supplement: Text S1 — Figure 1: Distribution of correlation scores for the Fe/Mn-SODs protein family. Figure 2: Correlation score spread (in logarithmic scale) for Fe/Mn-SODs (black) and 1000 alignments generated by shuffling the columns from the Fe/Mn-SODs alignment (red).Figure 3: Correlation score spread for 1000 shuffled alignments for (A) Peroxidases-catalases and (B) C-type lysozymes/lactalbumins. (DOC) [file pone.0027786.s020.doc]

# Using amino acid correlation and community detection algorithms to identify functional determinants in protein families

Lucas Bleicher, Ney Lemke, Richard Charles Garratt

***Control procedure***

If we calculate all correlation scores for amino acid pairs whose occurrence is above the minimum sub-alignment threshold, it is possible to obtain score histograms for protein families, as showin in Figure 1:

**Figure 1: Distribution of correlation scores for the Fe/Mn-SODs protein family.**

As can be seen in the histogram, most pairs have a near-zero absolute score (i.e., they are completely uncorrelated), but the curve decreases smoothly, with some pairs still presenting high correlation scores (up to 83).

To estimate the correlation score values that could be observed due to frequency bias and alignment size, the following procedure is applied. One thousand alignments were generated by shuffling each column of the original alignment. The result will be an alignment with exactly the same size and positional amino acid distribution, but it is expected to lose the correlation information obtained by the method. The occurrence of each possible absolute score was counted for each alignment and then averaged for the total number of alignments. The result can also be shown as an histogram, using error bars to denote standard deviation for the occurrence of a given correlation score for all 1000 alignments. Such histogram for Fe/Mn-SODs is shown in figure 2 along with the same data obtained from the original alignment:


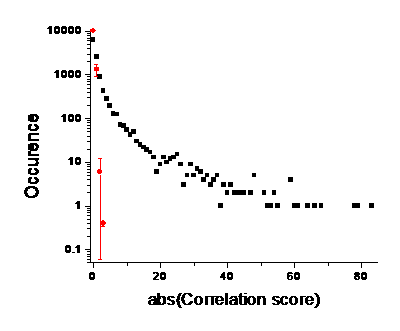


**Figure 2: Correlation score spread (in logarithmic scale) for Fe/Mn-SODs (black) and 1000 alignments generated by shuffling the columns from the Fe/Mn-SODs alignment (red).**

This histograms shows that virtually all pairs have a score of zero or one, with very few pairs producing scores of 2-4.

Similar results were obtained for the other protein families in this study, as shown in figure 2.


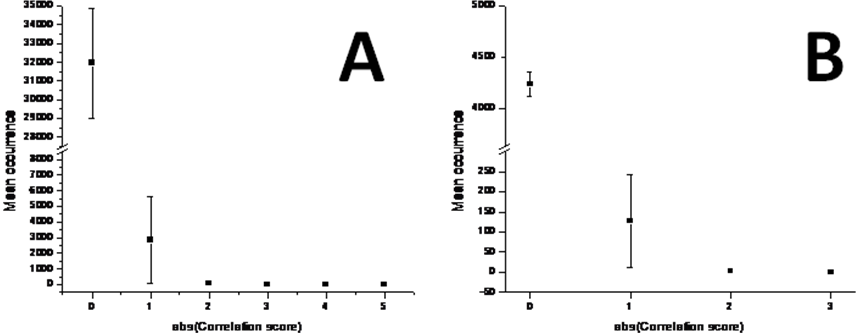


**Figure 3: Correlation score spread for 1000 shuffled alignments for (A) Peroxidases-catalases and (B) C-type lysozymes/lactalbumins**
