# Supplementary material for: Using Amino Acid Correlation and Community Detection Algorithms to Identify Functional Determinants in Protein Families
Source: PLoS One. 2011 Dec 20;6(12):e27786. doi: 10.1371/journal.pone.0027786 (PMC3243672; doi:10.1371/journal.pone.0027786)
Supplement: Text S2 — Table 1: Network decomposition for McBASC, cutoff = 0.5maximum. Table 2: Network decomposition for McBASC, cutoff = 0.8maximum. Table 3: Network decomposition for mutual information, cutoff = 0.8maximum. Table 4: Network decomposition for ELSC, cutoff = 0.8maximum. (DOC) [file pone.0027786.s021.doc]

# Using amino acid correlation and community detection algorithms to identify functional determinants in protein families

Lucas Bleicher, Ney Lemke, Richard Charles Garratt

**Results from other correlation analysis methods**

To exemplify the difference in outcome when using traditional correlation methods, we generated correlation data with McBASC (the original correlation metric proposed by Gobel *et al.* in 1994), Mutual Information (the leading method used nowadays for correlation studies, usually to find contact pairs) and ELSC, a perturbation-based method which uses explicit likelihood to calculate correlations (Dekker et al., 2004: A perturbation-based method for calculating explicit likelihood of evolutionary co-variance in multiple sequence alignments. *Bioinformatics* **20**: 1565-1572). Since all those methods will report a correlation value for each pair of positions, we have to apply a cutoff in order to generate networks. Two cutoffs (50% and 80% of the maximum correlation value found) were used for each method, and the results after community detection can be seen in the tables below.

| **Community 1** | | | | | | | | | | | | | | | | |
| --- | --- | --- | --- | --- | --- | --- | --- | --- | --- | --- | --- | --- | --- | --- | --- | --- |
| 122 | | 123 | | 161 | | 163 | | 164 | | 165 | | 168 | | | 173 | |
| **Community 2** | | | | | | | | **Community 3** | | | | | | | | |
| 28 | | 32 | | 36 | | 76 | | 24 | | | 121 | | | 148 | | |
| **Community 4** | | | | | **Community 5** | | | | **Community 6** | | | | **Community 7** | | | |
| 71 | 72 | | 145 | | 33 | | 80 | | 75 | | 127 | | 162 | | | 175 |

**Table 1: Network decomposition for McBASC, cutoff=0.5maximum**

| **Community 1** | | | | **Community 2** | |
| --- | --- | --- | --- | --- | --- |
| 161 | 164 | 165 | 168 | 28 | 76 |

**Table 2: Network decomposition for McBASC, cutoff=0.8maximum**

As can be seen above, if we apply a community detection procedure for correlation data obtained using McBASC with a 50% cutoff, we have communities related to oligomeric state (community 3) and metal affinity (community 4). However, we will have no information about the interdependence of these communities, nor the fact that, if we calculate amino acid specific information, as in our method, community 4 will decompose into two different groups as show in figure 1 in this article (due to *specific* amino acids in those positions determining metal specificity). With a stronger cutoff (0.8), we only have two communities with residues which are either very close in the sequence (community 1) or in the structure (residues 28 and 76 are in contact, as seen in PDB 3ESF).

For mutual information, using 50% as a cutoff produces a very large number of correlated positions which separates into two communities, one containing 87 positions and another containing 49 positions, which seem to be unrelated to the functional classes described for SODs (data not shown). A higher cutoff (80%) generates three small comunities, shown below, which are also unrelated to metal specificity or oligomeric states:

| **Community 1** | | | | **Community 2** | | | | **Community 3** | | |
| --- | --- | --- | --- | --- | --- | --- | --- | --- | --- | --- |
| 18 | 22 | 153 | 193 | 152 | 154 | 199 | 200 | 151 | 205 | 206 |

**Table 3: Network decomposition for mutual information, cutoff=0.8maximum**

The ELSC method also generates a very high number of correlated positions with a cutoff of 50% for the maximum observed value, with nine communities composed of 25, 19, 17, 12, 2, 2, 2 and 2 positions, respectively (data not shown), while the more stringent .8 cutoff results in seven smaller communities, shown below:

| **Community 1** | | | | | | | | | | | | **Community 2** | | | | | | | |
| --- | --- | --- | --- | --- | --- | --- | --- | --- | --- | --- | --- | --- | --- | --- | --- | --- | --- | --- | --- |
| 50 | | 52 | | 54 | | 55 | | 57 | | 74 | | 53 | | 56 | | 129 | | 190 | |
| **Community 3** | | | | | **Comm. 4** | | | | **Comm. 5** | | | | **Comm. 6** | | | | **Comm. 7** | | |
| 46 | 48 | | 49 | | 64 | | 120 | | 67 | | 120 | | 145 | | 146 | | 174 | | 176 |

**Table 4: Network decomposition for ELSC, cutoff=0.8maximum**

Usage of the previously mentioned methods can be useful for the search of contact pairs when only pairwise information is considered: for McBASC and ELSC, for example, the highest correlated pairs correspond to a close contact (27-76 for McBASC and 56-74 for ELSC), but the lack of specific amino acid information make them incomplete to the kind of application we propose – the search for function related groups of amino acids in protein family sub-classes.

The same protein family has been studied previously by our group with the Statistical Coupling Analysis (SCA) method (Bachega et al., 2009: Systematic structural studies of iron superoxide dismutases from human parasites and a statistical coupling analysis of metal binding specificity. Proteins 77: 26-37). By that time, the definition of SCA was partially residue specific: correlation was defined as the overall shift in residue frequencies in a position upon the presence of a given residue in another position. Therefore, there was a partial information about residue type, but lacking the possibility of analyzing anti-correlation. While, that initial formulation of SCA was still able to identify groups of residues related to metal specificity and oligomeric state, from 2009 onwards the authors of SCA adopted a new formulation which abandons residue specificity and only reports an overall correlation measure between two positions (Halabi et al., 2009: Protein sectors: evolutionary units of three-dimensional structure. Cell 138:774-786).
